# Characterization of Paraquat-Induced miRNA Profiling Response in hNPCs Undergoing Proliferation

**DOI:** 10.3390/ijms151018422

**Published:** 2014-10-13

**Authors:** Min Huang, Dan Lou, Qian Cai, Xiuli Chang, Xinjin Wang, Zhijun Zhou

**Affiliations:** 1 School of Public Health/MOE Key Lab of Public Health Safety/WHO Collaborating Center for Occupational Health, Fudan University, Shanghai 200032, China; E-Mails: clever_hmin@aliyun.com (M.H.); delicia.ldan@gmail.com (D.L.); xlchang@shmu.edu.cn (X.C.); 12211020063@fudan.edu.cn (X.W.); 2 Department of Occupational and Environmental Health, School of Public Health, Ningxia Medical University, Yinchuan 750000, China; E-Mail: caiqian1212@126.com; 3 Shanghai Municipal Center for Disease Control & Prevention, Shanghai 200336, China

**Keywords:** paraquat, hNPCs, miRNA profiling, developmental neurotoxicity, target genes, biological processes, pathways

## Abstract

Aberration during the development of the central nervous system (CNS) due to environmental factors underlies a variety of adverse developmental outcomes. Paraquat (PQ) is a widely studied neurotoxicant that perturbs the normal structure/function of adult CNS. Yet, the impacts of PQ exposure on the developing CNS remain unclear. miRNAs represent a class of small non-coding RNA molecules involved in the regulation of neural development. Thus in the present study, we analyzed the impacts of PQ on the miRNome of human neural progenitor cells (hNPCs) during proliferation by using the Exiqon miRCURY™ LNA Array. A total of 66 miRNAs were identified as differentially expressed in proliferating hNPCs upon PQ treatment. miRTarBase prediction identified 1465 mRNAs, including several genes (e.g., *nestin*, *sox1*, *ngn1*) previously proved to be associated with the neural proliferation and differentiation, as target genes of PQ-induced differentially expressed miRNAs. The database for annotation, visualization and integrated discovery (DAVID) bioinformatics analysis showed that target genes were enriched in regulation of cell proliferation and differentiation, cell cycle and apoptosis as well as tumor protein 53 (p53), Wnt, Notch and mitogen-activated protein kinases (MAPK) signaling pathways (*p* < 0.001). These findings were confirmed by real-time RT-PCR. Based on our results we conclude that PQ-induced impacts on the miRNA profiling of hNPCs undergoing proliferation may underlie the developmental neurotoxicity of PQ.

## 1. Introduction

Paraquat (1,1'-dimethyl-4,4'-bipyridinium, PQ), is a widely used non-selective herbicide, defoliant and desiccant with a well-known high efficiency. PQ absorbed orally was found to be persistent in brain with an apparent half-life of approximately 4 weeks and was cumulative with a linear pattern [1]. Due to the structural similarity with neurotoxicant MPP^+^ (1-methyl-4-phenylpyridinium ion) and the large induction of oxidative stress, the neurotoxicity of PQ in human as well as animal models has been generally accepted [2,3,4]. However, the putative adverse effects of PQ on the developing brain have rarely been investigated to date. Direct evidence exists that PQ was able to cross the placenta and accumulated to much higher level than in maternal blood [5]. The developing human brain is uniquely vulnerable to toxic chemical exposures, and major windows of developmental vulnerability occur in utero and during infancy and early childhood [6]. During these sensitive life stages, PQ might cause permanent brain injury at low levels of exposure that would have little or no adverse effect in an adult [6].

The mammalian central nervous system (CNS) consists of multiple neuronal cell types, as well as glial cells, astrocytes and oligodendrocytes. All these cells are all derived from common neural precursor cells (NPCs, also known as neural stem cells (NSCs)) and organized in a limited space within a particular time period during neurogenesis, which is closely related to the normal initiation from NPCs [7]. Different transcription factors (TFs) and signaling pathways have been demonstrated as crucial contributors to the intricate gene expression regulatory networks involved in NPC proliferation [8]. Likewise, epigenetic and gene expression regulated by non-coding RNAs especially microRNAs (miRNAs) have been described as additional and essential regulatory mechanisms for NPC maintenance [9].

miRNAs are small, non-coding RNA molecules of 21–25 nucleotides in length that regulate gene expression by base-pairing with the transcripts of their targets, *i.e.* protein-coding genes, leading to down regulation or repression of the target genes [10]. More than 50% of all identified miRNAs are expressed in the brain, where they play a particularly significant role in neural development by regulating a wide range of cellular processes, including cell proliferation, metabolic processes, apoptosis, and stress responses, at the post-transcriptional level [11]. PQ exposure may disrupt the timing and/or spatial regulation of these miRNAs, and give rise to a series of neurodevelopmental disorders [6].

During the last two decades, studying chemical disturbances of human neural progenitor cells (hNPCs) has been established as an alternative to the *in vitro* testing approach for the identification of potential developmental neurotoxicants [12]. Our previous study has demonstrated that PQ triggered reactive oxygen species (ROS) related apoptosis in hNPCs undergoing proliferation [13]. However, the mechanisms responsible for PQ-induced apoptosis have not been well understood. Thus in the present study, the miRNA expression profiling of hNPCs was chosen as one of the toxicological molecular endpoints. We explored the potential biological function of the observed differentially expressed miRNAs by comprehensively analyzing their target genes using a systems-based approach. Several biological processes and pathways were identified as related to PQ exposure. Our results not only indicate that PQ impacts, in a dose-dependent manner, genes and pathways previously identified to be critical for neural development, but also propose new targets to be further investigated in PQ-induced developmental neurotoxicity.

## 2. Results and Discussion

### 2.1. Effects of Paraquat (PQ) on Human Neural Progenitor Cells (hNPCs) Viability

**Figure 1:**
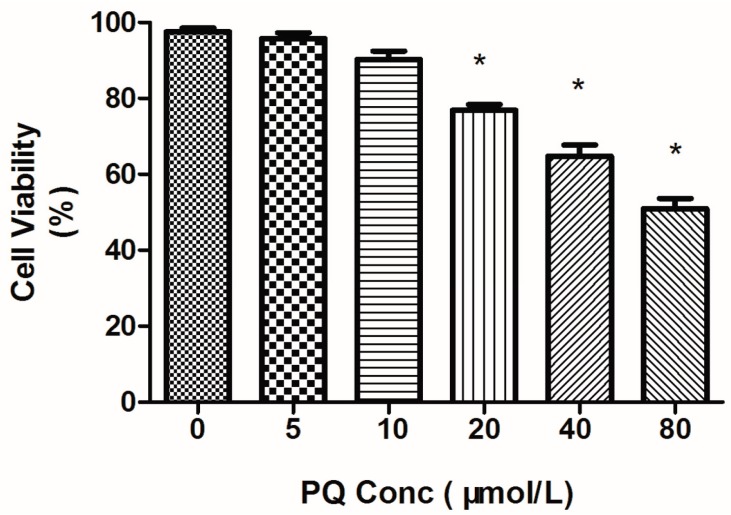
Paraquat (PQ)-induced dose-dependent decrease of cell viability in human nNeural progenitor cells (hNPCs). hNPCs were treated with PQ for 24 h and cell viability was measured by quantitative alamar blue assay. * Indicate statistically significantly lower than that in the control (*p* < 0.05). *n* = 3 independent biological replicates for alamar blue assay.

The effects of PQ on viability of hNPCs were determined by alamar blue assay. When compared with the control group, cell viability was significantly affected after exposure to more than 20 μmol/L of PQ for 24h. It was noticed that cell viability decreased with the increase of PQ concentration. The surviving cells were reduced by 30.5% under 40 μmol/L PQ exposure and were reduced to 51.8% upon 80 μmol/L PQ treatment (Figure 1).

### 2.2. Effects of PQ on hNPCs Apoptosis

**Figure 2:**
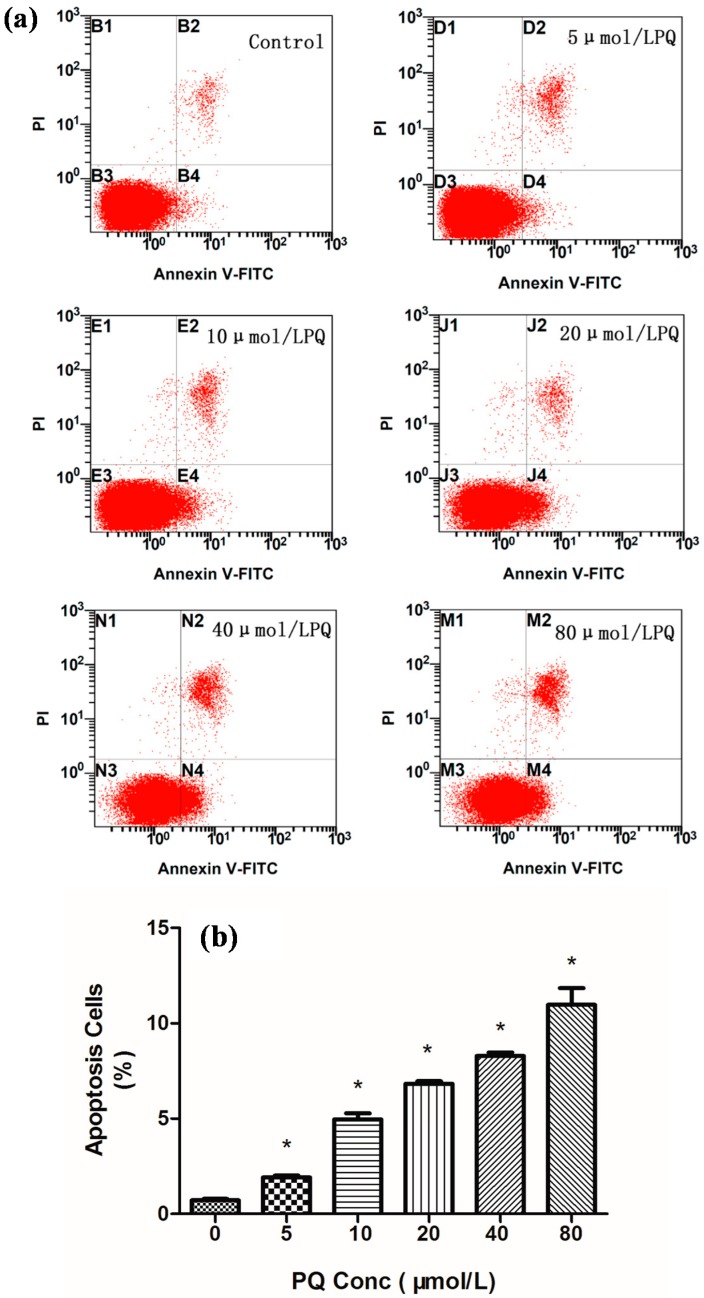
PQ-induced a dose-dependent increase of apoptosis shown in dot plot (**a**) and the percentage (**b**) in hNPCs. hNPCs were treated with PQ for 24 h and apoptotic cells were identified and quantified using the Annexin V-FITC apoptosis detection kit. Dot-plot (**a**) shows hNPCs staining with Annexin V-FITC *versus* propidium iodide (PI) resulting in distinct population. Cells staining with Annexin but without PI staining (lower right quadrant) can be considered as apoptotic cells and the percentage of apoptotic cells was calculated (**b**). * Indicate statistically significantly increase as compared to that in the control (*p* < 0.05). *n* = 3 independent biological replicates for flow cytometric analysis.

To examine whether cell apoptosis was involved in the PQ-induced cytotoxicity, we performed flow cytometric analysis of PQ-treated hNPCs. Figure 2 showed that PQ treatment increased the percentage of apoptotic cells at concentrations greater than 5 μmol/L. A clear dose-response relationship has been demonstrated between the percentage of apoptotic cells and the PQ concentration by trend test (*p* < 0.05).

### 2.3. Effects of PQ on miRNA Profiling of hNPCs

**Figure 3:**
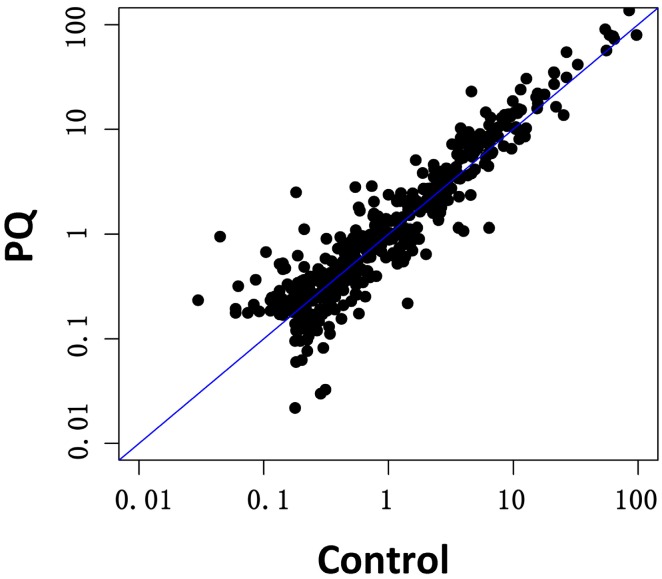
miRNA expression profiles were altered after PQ treatment for 24 h. Total RNAs including miRNAs were prepared from freshly isolated control hNPCs (without PQ treatment) and PQ-treated hNPCs (20 μmol/L, 24 h). miRNA expression profiles in these cells were analyzed using Exiqon miRCURY™ LNA Arrays containing capture probes targeting all miRNAs for human, mouse or rat registered in the miRBase version 18.0 at the Sanger Institute. A scatter plot of the microarray is presented.

As a next step, hNPCs were used to determine the effects of PQ on miRNA expression. According to the results from cell viability assays and apoptosis measurement, 20 μmol/L PQ upon which the viability and apoptosis percentage of hNPCs were significantly mildly affected was chosen to treat hNPCs for miRNA profiling. We analyzed the global changes in miRNA profiles in PQ-treated (20 μmol/L, 24 h) cells using Exiqon miRCURY™ LNA Arrays. In addition to the data presented herein, we performed two replicates of miRNA microarray (based on miRBase Release 16.0) with similar results. Differentially expressed miRNAs are summarized in Table 1. The exposure to PQ led to significant alterations (fold change (FC) > |2.0|, *p* < 0.05) in the expression of 66 miRNAs (Figure 3).

From 66 miRNAs that responded to PQ exposure, 40 were up-regulated 2- to 21-fold, while 26 were down-regulated up to 100-fold (Table 1). miRNAs involved in embryonic and adult neurogenesis such as *hsa-mir-124*, *hsa-mir-10a*, or *hsa-let-7 family* were found significantly dysregulated by PQ. Known tumor-suppressor *hsa-mir-34c*, which was recently found to be involved in brain development and spermatogenesis [14], was significantly upregulated in hNPCs treated with PQ. Futher, *hsa-mir-491*, which is involved in neurosteroidogenesis and pathogenesis of multiple sclerosis [15], was found significantly downregulated by PQ.

**Table 1:** Listing of differentially expressed miRNAs in the microarray analysis.

microRNA	Fold Change	microRNA	Fold Change
Up-Regulated		Down-Regulated	
*hsa-miR-550a-5p*	21.36	*hsa-miR-3182*	0.01
*hsa-miR-550b-2-5p*	13.81	*hsa-miR-4308*	0.11
*hsa-miR-378b*	7.92	*hsa-miR-3679-3p*	0.11
*hsa-miR-4784*	6.50	*hsa-miR-145-3p*	0.12
*hsa-miR-3142*	5.30	*hsa-miR-1273g-3p*	0.15
*hsa-let-7f-5p*	5.19	*hsa-miR-124-3p*	0.18
*hsa-miR-3175*	4.99	*hsa-miR-3178*	0.27
*hsa-miR-4446-5p*	4.29	*hsa-miR-3675-3p*	0.27
*hsa-miR-4657*	3.91	*hsa-miR-1184*	0.30
*hsa-miR-4291*	3.89	*hsa-miR-299-5p*	0.31
*hsa-miR-1304-5p*	3.70	*hsa-miR-491-3p*	0.32
*hsa-miR-378d*	3.35	*hsa-miR-4456*	0.32
*hsa-miR-4275*	3.27	*hsa-miR-208a-5p*	0.33
*hsa-miR-10a-5p*	3.26	*hsa-miR-574-3p*	0.33
*hsa-miR-195-5p*	3.19	*hsa-miR-589-5p*	0.34
*hsa-miR-4289*	3.12	*hsa-miR-5196-3p*	0.37
*hsa-let-7d-5p*	3.06	*hsa-miR-371b-3p*	0.39
*hsa-miR-412-3p*	2.99	*hsa-miR-3646*	0.39
*hsa-miR-34c-5p*	2.86	*hsa-miR-1284*	0.42
*hsa-miR-4484*	2.84	*hsa-miR-1321*	0.44
*hsa-miR-98-5p*	2.72	*hsa-miR-4524b-5p*	0.44
*hsa-miR-320d*	2.67	*hsa-miR-4521*	0.45
*hsa-miR-5586-5p*	2.56	*hsa-miR-4305*	0.45
*hsa-miR-27a-3p*	2.43	*hsa-miR-498*	0.46
*hsa-miR-3654*	2.40	*hsa-miR-944*	0.47
*hsa-miR-3686*	2.39	*hsa-miR-519e-5p*	0.48
*hsa-miR-3135a*	2.37		
*hsa-miR-378e*	2.32		
*hsa-miR-320e*	2.30		
*hsa-let-7g-5p*	2.23		
*hsa-miR-30c-5p*	2.19		
*hsa-miR-1290*	2.17		
*hsa-miR-30c-1-3p*	2.16		
*hsa-miR-484*	2.15		
*hsa-miR-23a-3p*	2.10		
*hsa-miR-647*	2.09		
*hsa-miR-4742-3p*	2.08		
*hsa-miR-23c*	2.05		
*hsa-miR-767-5p*	2.05		
*hsa-miR-376a-3p*	2.04		

### 2.4. Functional and Pathway Analysis

To gain insight into the biological significance of the global or system-level impacts of PQ on hNPCs, 1465 mRNAs were identified as target genes of PQ-induced differentially expressed miRNAs through miRTarBase prediction. We further evaluated enriched functional annotation categories (Gene Ontology and Kyoto encyclopedia of genes and genomes (KEGG)) for these target genes using the database for annotation, visualization and integrated discovery (DAVID) bioinformatics tools according to their enrichment *p*-value.

**Figure 4:**
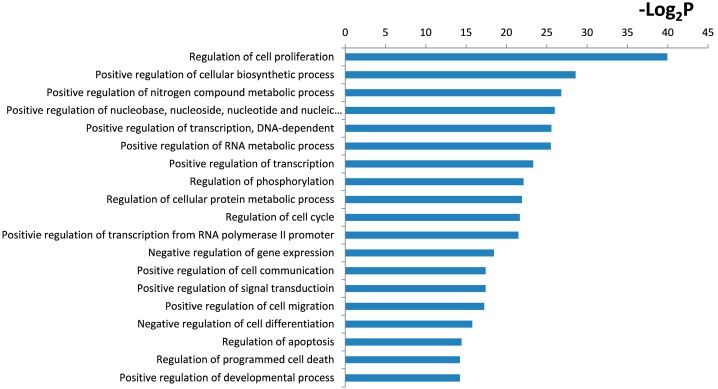
Gene Ontology (GO) analysis within the target genes of significantly altered miRNAs after PQ exposure was performed using the database for annotation, visualization and integrated discovery (DAVID) bioinformatics tools. The enriched GO biological processes were identified and listed according to their enrichment *p*-value (*p* < 0.005).

**Figure 5:**
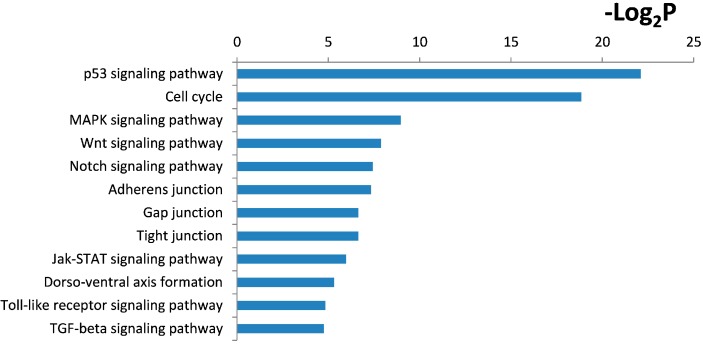
Kyoto encyclopedia of genes and genomes (KEGG) pathway analysis within the target genes of significantly altered miRNAs after PQ exposure was performed using DAVID bioinformatics tools. The enriched KEGG pathways were identified and listed according to their enrichment *p*-value (*p* < 0.005).

Gene Ontology cluster analysis showed that these target genes of PQ-induced differentially expressed miRNAs are enriched in regulation of cell proliferation, transcription, positive regulation of cellular biosynthetic process, regulation of transcription, regulation of cell cycle and apoptosis (all categories with *p* < 0.001) (Figure 4). KEGG pathway cluster analysis showed that the target genes are related to tumor protein 53 (p53), Wnt, Notch and mitogen-activated protein kinases (MAPK) signaling pathways (Figure 5).

### 2.5. Real-Time RT-PCR Validation of PQ-Responsive miRNAs and Target Genes

Real-time RT-PCR was performed to validate the microarray data. To identify the toxic effects of PQ on developing CNS, we chose a set of neural-specific miRNAs as our targets. As mentioned above, miRNAs such as *hsa-mir-124, hsa-mir-10a*, *hsa-mir-34c, hsa-mir-9, hsa-mir-34c and hsa-let-7* family were previously proved to be involved in embryonic/adult neurogenesis during brain development [14].

**Figure 6:**
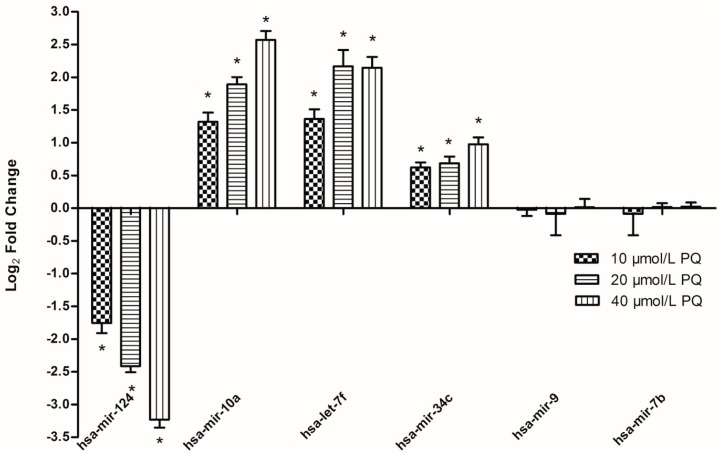
PQ-induced miRNA expression alterations. The selected miRNAs include three upregulated miRNAs, one downregulated miRNA and two unaffected miRNAs. hNPCs were treated with 0, 10, 20, or 40 μmol/L PQ for 24 h and miRNA were isolated for real-time RT-PCR analysis. Data represent means ± SE with each analysis performed in triplicate. All values showed in the histograms represented as log_2_ fold change compared to the control group average of 0. * Indicate statistically significantly higher than that in the control group (*p* < 0.05). *n* = 3 independent biological replicates for real-time RT-PCR.

According to the results from the cell viability assay and apoptosis measurement, the concentration of 10, 20, 40 μmol/L PQ were chosen to explore the dose-response relationship. An Exiqon microRNA assay was used to analyze the expression of these miRNAs after treatment with 0, 10, 20, 40 μmol/L PQ for 24 h. Using independent procedures, we could confirm the microarray data (Figure 6), that is, a clear concentration-dependent induction of hNPCs differentiation-related miRNA (*hsa-mir-10a, hsa-let-7f* and *hsa-mir-34c*) and a significant down-regulation of the expression of hNPCs proliferation-maintained miRNA (*hsa-mir-124*) in comparison to the solvent control. We did not observe any changes in the expression of *hsa-mir-9* and *hsa-let-7b* in our microarray data, so these miRNAs were included in the RT-PCR validation as negative control.

**Figure 7:**
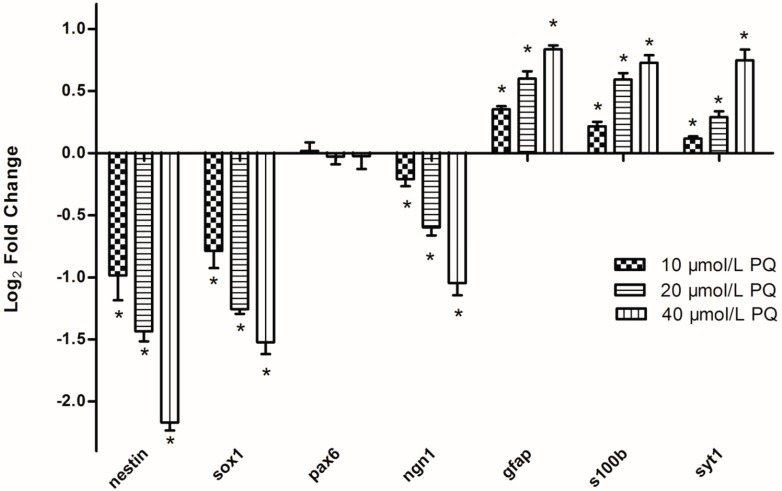
PQ-induced mRNA expression alterations. These genes selected from 1,465 targets of PQ-induced differentially expressed miRNA were known to be involved in the hNPCs proliferation and differentiation. hNPCs were treated with 0, 10, 20, or 40 μmol/L PQ for 24 h and mRNA were isolated for real-time RT-PCR analysis. Data represent means ± SE with each analysis performed in triplicate. All values showed in the histograms are represented as log_2_ fold change compared to the control group average of 0. * Indicate statistically significantly higher than that in the control group (*p* < 0.05). *n* = 3 independent biological replicates for real-time RT-PCR.

In order to verify the target prediction and functional analysis by real-time RT-PCR, we selected genes known to be involved in the hNPCs proliferation and differentiation (neurofilament (*nestin)*, sex determining region Y-box 1 (*sox1*), paired box protein (*pax6*), neurogenins (*ngn1*), glial fibrillary acidic protein (*gfap*), S 100 calcium binding protein B (*s100b*), synaptotagmin-1 (*syt1*) from the 1465 targets. After exposure to different concentrations of PQ for 24 h, a series of dose-dependent depression of neural genes (*sox1, ngn1* and *nestin*) were observed, whereas the expression of neuronal genes (*syt1*) and astrocytic genes (*gfap, s100b*) were up-regulated (Figure 7).

### 2.6. Discussion

PQ is a widely characterized neurotoxicant able to induce a series of nervous system disorders, including neurobehavioral defects and neurodegenerative diseases such as Parkinson’s disease [16,17]. Although there is supporting evidence that PQ can cross the placental barrier and persist in the brain with a half-life of 28 days, the impacts of PQ on the developing brain are rarely investigated. Thus in this study, we investigated the dose-dependent impact of PQ on genomic miRNA expression within hNPCs exposed during proliferation. Using microarray technology and systems-based approach, we identified the effects of PQ on the expression of miRNAs and mRNAs associated with biological processes/pathways, including cell proliferation, cellular biosynthetic process, cell cycle, transcription regulation, Wnt signaling, Notch signaling, p53 signaling, and MAPK signaling pathway. PQ-induced dose-dependent miRNA expression alteration were further confirmed at the level of mRNA by demonstrating the induction of hNPCs differentiation related genes and the accompanied inhibition of genes involved in hNPCs proliferation (Figure 6 and Figure 7).

To our knowledge, this is the first comprehensive analysis of miRNAs in hNPCs under PQ exposure. Although very little is known about the role of miRNAs in PQ-treated hNPCs, there have been several studies examining disturbed miRNAs profiling by other chemicals [12]. Many of the miRNAs, which had been reported to be altered by other chemicals, were also altered during the PQ exposure in this study.

miRNA target prediction and further functional analysis reveals that PQ-altered miRNAs are related to the regulation of proliferation and differentiation of hNPCs. *mir-124* and *mir-9*, the two most abundant miRNAs in the CNS, showed different responses to PQ treatment: while *mir-124* was down-regulated after PQ exposure, no significant effect on *mir-9* expression could be observed after PQ exposure. *mir-124* has been proposed to promote neuronal differentiation by targeting multiple anti-neuronal factors while *mir-9* was shown to maintain the proliferation of neural progenitors [18]. Over-expression of *mir-124* in mouse embryonic carcinoma (P19) cells promotes neurite extension by altering the level or localization of cell division control protein 42 homolog gene (*Cdc42*) and ras-related C3 botulinum toxin substrate 1 gene (*Rac1*), the activation of which attenuates neurite outgrowth [19]. The loss of *mir-9* function suppresses the proliferation and promotes the migration of human embryonic neural progenitors by targeting stahmin, which increases microtubule instability in migrating neuroblasts [20].

The *let-7* miRNA family including *let-7d*, *let-7f* and *let-7g* were up-regulated after PQ exposure, while *let-7b* and *let-7e* expression were not significantly altered. It is reported that the *let-7* family was enriched in both embryonic and adult brains [21] and the expression of *let-7* is upregulated during NSC specification. *Let-7b* was demonstrated to regulate the proliferation and self-renewal of NPCs by targeting high mobility group-AT-hook 2 (Hmga2), which promotes the NPC self-renewal in the central and peripheral nervous system of fetal and young mice [18,22]. Interestingly, although rarely reported in hNPCs research, *let-7f* was found to negatively regulates hepatic differentiation of human adipose tissue-derived stem cells [23], which might imply the role of *let-7f* in regulating hNPCs proliferation and differentiation. In addition, the increased expression of *let-7d* and *let-7g* in hNPCs after PQ exposure also provide potential targets for PQ neurotoxicity.

*Mir-10a* is highly evolutionarily conserved and localized with homeotic (*Hox*) gene clusters. *Hox* genes are conserved transcription factors, regulating the anterior-posterior pattern formation during development and have been implicated in neural tube closure [24]. *Mir-10a* was significantly induced during neural proliferation under PQ exposure in our hNPCs system. *Mir-10a* is up-regulated in response to retinoic acid, a common inducer of cellular differentiation in various cell types and was demonstrated to contribute to retinoic acid-induced differentiation of neuroblastoma cells [25]. In addition, it is reported that *mir-34c* is a target of p53 and plays a role in control of cell proliferation and adhesion-independent growth [14]. The results from our study suggest that PQ affects neural proliferation and differentiation processes are specific for *mir-124*, *mir-10a*, *let-7d/f/g* and *mir-34c* but not *mir-9*.

The quantification of neural proliferation/differentiation related genes using real time RT-PCR further confirmed the impacts of PQ on these miRNAs. After exposure to PQ for 24 h, neural genes (*Sox1*, *pax6*, *ngn1* and *nestin*) supposed to express in normal hNPCs were suppressed, whereas neuronal genes (*syt1*) and astrocytic genes (*gfap*, *s100b*) supposed to be repressed in normal hNPCs were activated. These results suggest that PQ exposure suppresses the normal proliferation of hNPCs and promotes abnormal differentiation at improper timing, which may results in serious neurodevelopmental disorders.

The KEGG pathway analysis using DAVID revealed that wnt and notch signaling pathway were also involved in the PQ-induced miRNA profiling alteration of hNPCs. Wnt and notch signaling pathways are critical for normal morphogenesis and development because they regulate NPCs differentiation in the early developing embryo [26]. As shown in Figure 5, the wnt and notch signaling pathway were observed to be altered by PQ exposure, suggesting a significant impact of PQ on the proliferation and differentiation of hNPCs.

The impacted proliferation and improper differentiation of hNPCs induced by PQ exposure were demonstrated by our previous study [13]. In addition, the alterations of ROS, p53, cyclin-dependent kinase inhibitor 1 (p21), and metallothionein-3 (MT3) involved in p53 and the MAPK signaling pathway were also observed in our previous study, which to some extent confirmed the pathway analysis in this study.

In the present study, we have identified several molecular targets (both miRNAs and mRNAs) and processes that PQ might disrupt in a dose-dependent manner including signaling pathways that have been previously identified to be involved in the proliferation or differentiation of hNPCs. This whole genomic miRNA dataset reinforces the notion that PQ might mediate developmental neurotoxicity by disrupting the correct timing of proliferation and differentiation of neural progenitor cells. The functional and pathway analysis also highlights the need of future research on the cross-talk and intersections of these key processes and pathways, in order to further elucidate the underlying neurodevelopmental effects of PQ and similar metallic compounds on the developing organism.

## 3. Experimental Section

### 3.1. Chemicals and Reagents

Paraquat dichloride (molecular weight 257.16, analytical standard, PQ) was obtained from Sigma-Aldrich (St. Louis, MO, USA). Dosing solutions were prepared by dissolving the calculated amount of PQ in cell culture medium, following the approved standard operating procedures for handling toxic agents. All other reagents were obtained from commercial sources and were of the highest available grade.

### 3.2. Cell Culture and Treatments

Immortalized human neural progenitor cells (hNPCs, ReNcell CX cells) were purchased from Millipore (Temecula, CA, USA). Cells frozen at passage two were thawed and expanded in ReNcell NSC Maintenance Medium supplemented with fresh epidermal growth factor (EGF) (20 ng/mL), fibroblast growth factor 2 (FGF-2) (20 ng/mL) and penicillin-streptomycin (100 U/mL) in dishes or plates coated by laminin (Sigma-Aldrich, St. Louis, MO, USA). Cells were incubated in a humidified atmosphere with 5% CO_2_ at 37 °C and then passaged by accutase at about 80% confluence. In present study, cells from passage seven to passage nine were utilized for all experiments.

Prior to being used in experiments, the expression of Nestin was determined to confirm the neural progenitor nature of these hNPCs as previously described [13]. Confluent cells treated with 0, 5, 10, 20, 40, or 80 μmol/L PQ respectively for 24 h were collected for subsequent experiments. Cells treated with 20 μmol/L PQ (based on the cell viability and apoptosis assay) for 24 h were used for miRNA microarray analysis. All experiments were repeated at least three times for each treatment group.

### 3.3. Cell Viability Assay

The hNPCs viability was determined by alamar blue assay (Invitrogen, Camarillo, CA, USA). Following a 20 h PQ exposure, 10 μL alamar blue was added to each well and the 96-well plates were incubated at 37 °C for 4 h. Absorbance of alamar blue was detected at 570 nm using a spectrophotometer (EL × 800™ Absorbance Microplate Reader, BioTek, Colchester, VT, USA). Cell viability was obtained as a percentage of the value of survival cells in the control groups.

### 3.4. Cell Apoptosis Assay

Apoptotic cells were identified and quantified by double staining with Annexin V-FITC and propidium iodide (PI) using the Annexin V-FITC apoptosis detection kit (Major Biotech, Shanghai, China). Cells were plated in 6-well plates (1.0 × 10^5^ cells/mL) and treated with PQ of different concentrations for 24 h. For flow cytometric (FCM) analysis, the cells were harvested by centrifugation and washed with phosphate saline buffer (PBS). The cells were then re-centrifuged and re-washed with PBS twice and suspended in binding buffer at the concentration of 1.0 × 10^6^ cells/mL. Then 5 μL Annexin V-FITC and 10 μL PI were added into the cell suspension and incubated for 15 min in the dark at room temperature. Finally, 400 μL PBS was added to each sample and FCM analysis was performed with a flow cytometer (Beckman-Coulter Inc., Brea, CA, USA).

### 3.5. RNA Isolation

hNPCs treated with different concentrations of PQ for 24 h were harvested for the isolation of miRNA and mRNA. Cells cultured in the same medium without PQ were used as the negative control. Qiagen’s miRNeasy mini kit (Qiagen, Hilden, Germany) was used for miRNA isolation while RNeasy Plus mini kit (Qiagen, Hilden, Germany) was used for mRNA isolation. RNA quality and quantity were measured using the NanoDrop 1000 (Thermo Fisher Scientific, Waltham, MA, USA), and RNA integrity was determined by denaturing Agarose Gel (BioRad Inc., Berkeley, CA, USA) electrophoresis.

### 3.6. miRNA Microarray Analysis

The 7th generation of Exiqon miRCURY™ LNA Array (Exiqon, Vedbaek, Denmark), which contains capture probes targeting all miRNAs for human, mouse or rat registered in the miRBase version 18.0 (Exiqon, Vedbaek, Denmark) at the Sanger Institute, was used to quantify the genome-wide miRNA expression profile of hNPCs. miRNA samples collected from control hNPCs (without PQ treatment) and PQ-treated hNPCs (20 μmol/L, 24 h)were labeled using the miRCURYTM Hy3TM/Hy5TM Power labeling kit (Exiqon, Vedbaek, Denmark) and hybridized on the arrays. Following the washing steps, the slides were scanned using the Axon GenePix 4000B microarray scanner (Axon, Gilze, The Netherlands). Intensity values were extracted from scanned images using GenePix Pro 6.0 software (Axon, Gilze, The Netherlands) for all probes included on the arrays. Two independent biological replicates were performed. Raw intensities were normalized using Median normalization according to the averaged replicated miRNAs and miRNAs that intensities ≥30 in all samples. Student’s *t*-test was done to determine the statistical significance of the comparison between control hNPCs (without PQ treatment) and PQ-treated hNPCs (20 μmol/L, 24 h) Differentially expressed miRNAs were identified through Fold Change filtering (fold change (FC) > |2.0|, *p* < 0.05).

### 3.7. Target Prediction and Functional Analysis

The most dysregulated miRNAs after PQ exposure were selected for target prediction by using the miRTarBase (ISBLab, Hsinchu, Taiwan). The miRTarBase serves as an important repository for experimentally miRNA target interactions (MTIs), which are frequently updated by manually surveying research articles. The latest version (Release 4.5: 1 November 2013) was used [27].

To assess the function of all potential mRNA targets that were correlated with differentially expressed miRNAs, DAVID bioinformatics tools (DAVID Bioinformatics Resources 6.7, NIAID/NIH, Frederick, MD, USA) were used to identify enriched functional annotation categories. Entrez_gene_ID was used to upload the target gene list. Three classes of Gene Ontology (GO) and KEGG pathway terms were evaluated [28].

### 3.8. Real-Time RT-PCR Validation

To validate our findings, quantitative real-time PCR was performed to quantify miRNAs and mRNAs using Gene Amp PCR System 9700 (Applied Biosystems, Foster City, CA, USA). Complementary DNA (cDNA) was synthesized using Exiqon Universal cDNA synthesis kit (from miRNA, Exiqon, Vedbaek, Denmark) or ABI cDNA reverse transcription kit (from mRNA, Life Technologies, Carlsbad, CA, USA). Primers and probes were predesigned by the manufacturer. All PCR reactions were conducted according to the manufacturer’s instructions. Standard thermocycle conditions were used and all reactions were performed in triplicate. 2^−ΔΔ*C*t^ method was used to quantify the relative expression of each miRNA/mRNA. U6 and GAPDH were used as the housekeeping control of miRNA and mRNA respectively.

### 3.9. Statistical Analysis

Data was analyzed using Stata statistical software version 11.0 (Stata Corporation, College Station, TX, USA); GraphPad Prism (GraphPad Software, Inc., La Jolla, CA, USA) and expressed as mean ± standard error (SE). Statistical comparisons among different treatment groups were performed by student’s *t*-test, one-way analysis of variance (ANOVA) or Kruskal–Wallis Test. Bonferroni-corrected *post hoc* tests were conducted to adjust the observed significant level for multiple comparisons if the null hypothesis was rejected. Cuzick’s test for trend was used to examine the dose-response relationship between different dose groups. Differences with a *p*-value less than 0.05 were considered to be statistically significant.

## 4. Conclusions

In this study, we have identified several miRNAs and target genes in hNPCs during proliferation that PQ may disrupt in a dose-dependent manner. Altered miRNAs might play a particularly significant role in neural development by regulating at the post-transcriptional level in a wide range of cellular processes including cell proliferation, differentiation, apoptosis as well as stress responses. The dataset from our study may support the developmental neurotoxicity of PQ and reinforces the notion that PQ-mediated developmental neurotoxicity results from the disruption of a complex and varied set of processes. Future studies to examine the cross-talk and intersections of these targets and processes are needed to gain a better elucidation of the effects of PQ.
